# Transcriptome-based resistance analysis of susceptible and resistant tomato cultivars against *Meloidogyne incognita* infection

**DOI:** 10.3389/fpls.2026.1745106

**Published:** 2026-04-15

**Authors:** Zhao Mengxin, Wei Peiyao, Pan Song, Chen Zhijie, Peng Deliang, Ren Xianghui, Li Yingmei, Liu Chen

**Affiliations:** 1 Shaanxi Key Laboratory of Plant Nematology, Bio-Agriculture Institute of Shaanxi, Xi’an, China; 2 State Key Laboratory for Biology of Plant Diseases and Insect Pests, Institute of Plant Protection, Chinese Academy of Agricultural Sciences, Beijing, China; 3 Xi’an Jinpeng Seedling Co., Ltd, Xi’an, China

**Keywords:** differential gene expression, disease resistance, *Meloidogyne incognita*, root-knot nematode, transcriptome analysis

## Abstract

Tomatoes are widely cultivated worldwide and contribute substantially to vegetable production. However, the root-knot nematode (RKN) disease caused by *Meloidogyne incognita* poses a serious threat to tomato cultivation. The most economical and effective way to control this disease is to deploy resistant cultivars. While the disease resistance provided by the original *Mi-1* gene is gradually weakening, highlighting the need to identify new resistance genes for tomato breeding. In this study, RNA sequencing (RNA-seq) was performed on root samples from the resistant tomato cultivar 'Jinpeng M6' and the susceptible cultivar ‘Jinpeng No.1’, with sampling conducted at Stage 1 and 2 for ‘Jinpeng M6’ and Stage 1-6 for ‘Jinpeng No.1’ based on characterized *M. incognita* infection dynamics. Comparative transcriptome analysis identified differentially expressed genes (DEGs), and qRT PCR validated the expression patterns of 12 selected DEGs. A total of 9,673 DEGs were identified, with Stage 2 representing a critical period for distinguishing the infection-responsive patterns between the two cultivars. KEGG enrichment analysis revealed a dominant “Plant-pathogen interaction” pathway in resistant cultivar, while the susceptible cultivar showed enriched “Plant hormone signal transduction” and “Circadian rhythm - plant” pathways. qRT PCR confirmed DEG expression consistency with RNA-seq, and the shared phenylpropanoid biosynthesis pathway in both cultivars showed significant DEG expression divergence at Stage 2. The results indicate that the phenylpropanoid biosynthesis pathway exhibits cultivar-specific responses to nematode infection and potential roles in tomato resistance. This study provides a theoretical foundation forelucidating the molecular mechanism of tomato resistance to *M. incognita*, and a scientific basis for screening RKN-resistant tomato germplasm resources.

## Introduction

Tomatoes (*Solanum lycopersicum*) are highly valued for their nutritional benefits and delightful flavor (Lu and Zhu, 2022). They are cultivated extensively around the globe and play a crucial role in the agricultural economy (Zhu et al., 2018). In recent years, advancements in protected agriculture have ensured a consistent supply of tomatoes throughout the year. However, the long-term continuous cultivation has led to increasingly severe disease issues, with RKN disease posing a major threat (Seebold, 2014). This disease is primarily caused by *Meloidogyne incognita* (Jones et al., 2013). Once the second-stage juveniles (J2s) infect the plants, they establish feeding sites within the tomato roots and begin to develop. The infected roots develop characteristic root knots, which impede the plant’s ability to absorb nutrients and water, leading to stunted growth and poor overall development (Seebold, 2014). In severe cases, the entire plant may perish, resulting in yield losses of 30% to 50%, which can cause significant economic losses for tomato growers (Collange et al., 2011).

Currently, cultivating disease-resistant varieties is the most economical and effective method to control RKN disease (Fuller et al., 2008). In agricultural production practice, this strategy not only ensures yield but also reduces production costs and the use of chemical pesticides, aligning with the concept of sustainable development (El-Sappah et al., 2019). However, resistance genes specifically targeting RKN are extremely scarce in current tomato varieties. To date, only the *Mi-1* resistance gene has been applied in tomato resistance breeding. Nevertheless, affected by factors such as population variation of RKN and environmental conditions, the disease resistance ability of the *Mi-1* gene has been continuously weakened, posing significant potential risks to tomato cultivation (Vos et al., 1998). Therefore, it is highly urgent to explore more novel resistance-related genes through multiple approaches. Against this backdrop, modern molecular biology techniques can be applied to identify resistance-related clues from wild tomato germplasm, while gene editing technologies enable targeted modification of existing genes for RKN resistance enhancement. The implementation of these efforts must be based on an in-depth understanding of the interaction mechanism between tomatoes and RKN, as well as an accurate revelation of the resistance mechanism in tomatoes.

The interaction between plants and pathogens involves a process of two-way communication and mutual regulation. Once pathogens successfully infect plants, they can manipulate the plants’ metabolism and biological functions to create an environment conducive to their own growth and reproduction (Wang et al., 2022). On the other hand, when plants detect pathogen invasion, they carefully regulate the balance between immunity and development, activating various defense mechanisms to resist disease and protect themselves (Bai et al., 2022). The plant phenylpropanoid metabolic pathway is one of the most important component of plant disease and pest resistance mechanisms (Ninkuu et al., 2025). Research has confirmed that phenylpropanoid accumulation is a key factor contributing to the insect resistance of the wild tomato species, *S. habrochaites* (Wang et al., 2024). Multi-omics analyses have revealed the significance of phenylpropanoid metabolism in the defense response of lilies against *Fusarium* wilt (Deng et al., 2024). The cotton cultivar Pima-S6 resists infection by *F. oxysporum* f. sp. *vasinfectum* race 4 through enhancing phenylpropanoid metabolism (Ojeda-Rivera et al., 2024). As core products of the phenylpropanoid pathway, flavonoids exhibit significant genetic diversity, which is mainly attributed to transcriptional regulatory differences of key enzymes involved in flavonoid synthesis and regulation among different *Arabidopsis* ecotypes (Sun et al., 2015). The genetic diversity of phenylpropanoid metabolites in crops provides an important genetic basis for plant disease and pest resistance.

RNA sequencing (RNA-seq) is an essential tool for studying plant-pathogen interactions, particularly for analyzing gene expression, discovering functional genes, and identifying key pathways involved in disease resistance (Feng et al., 2020). Transcriptome sequencing data show that different plants respond to pathogen infections using multiple mechanisms, including the regulation of plant hormone expression, reactive oxygen species, signal transduction, and disease resistance-related genes (Heese et al., 2007). Previous studies have shown that certain genes undergo transcriptional changes in tomato varieties from regions such as India and the United States upon RKN infection (Schaff et al., 2007; Shukla et al., 2018). However, studies on the gene regulatory differences underlying the response of Chinese tomato cultivars to nematode infection based on transcriptome data remain relatively limited. Current domestic transcriptomic studies have mainly focused on the response of tomato salicylic acid (SA) pathway genes to the RKN MiPDCD6 protein, Mi-3-mediated hypersensitive response to *M. incognita* in *S. peruvianum*, and the roles of SA, jasmonic acid (JA), and reactive oxygen species in nematode resistance (Du et al., 2020a, 2020; Deng et al., 2023). To date, systematic comparative transcriptomic analysis between resistant and susceptible major cultivated tomato varieties in China in response to RKN infection are still lacking.

In the preliminary research conducted by our team, the tomato cultivar “Jinpeng No.1” was identified as a germplasm highly susceptible to RKN, while the tomato cultivar “Jinpeng M6” was identified as a germplasm highly resistant to *M. incognita* (unpublished data). This study aims to analyze the phenotypic differences between these two varieties after infection with RKN and use RNA-seq technology to mine differentially expressed genes (DEGs). It is expected to reveal the molecular mechanisms of susceptible and resistant tomatoes in response to RKN, improve the regulatory network of tomato resistance to RKN, and provide a theoretical basis for tomato disease-resistant breeding.

## Materials and methods

### Plant materials and nematode inoculation

In this study, we tested two tomato cultivars: the root-knot nematode-resistant cultivar “Jinpeng M6” and the nematode-susceptible cultivar “Jinpeng No.1”. The seeds were supplied by Xi’an Jinpeng Seedling Co., Ltd. To ensure sterility, the tomato seeds were soaked in 70% ethanol for 1 minute and then in 3% sodium hypochlorite for 10 minutes, followed by rinsing with sterile distilled water three times. From the third rinse, we took 250 μl of water to check for contamination, as described by Niu et al. (2017). After sterilization, the seeds were planted in 7-cm-diameter pots filled with sterilized sand soil, with one seed per pot. A total of 120 pots were established for each tomato cultivar, to ensure experiments of dynamic nematode development analysis, RNA isolation, and transcriptome sequencing of root tissues at successive developmental stages. The growth conditions were maintained at 16 hours of light and 8 hours of darkness, with temperatures of 26 ± 1 °C during the light period and 18 ± 1 °C during the dark period, and approximately 60% relative humidity. The *M. incognita* nematodes used in this study were continuously cultured on water spinach, provided by Professor Zhuokan from Huazhong Agricultural University. To prepare for the experiment, nematode egg masses were incubated in sterilized distilled deionized water (ddH_2_O) to hatch the J2s. The number of J2s hatched per unit volume was counted (Sudirman and Webster, 1995). When the tomato seedlings reached the four true-leaf stage, approximately 4 weeks after planting, we inoculated each plant with approximately 400 J2 RKNs by depositing the nematode suspension into four pre-drilled holes around the root circumference, followed by covering the inoculation sites with soil. The nematode inoculation dosage and method were performed according to the protocol described by Zhao et al. (2023).

### Acid fuchsin staining and nematode development analysis

Tomato root with nematodes samples were collected from 1 day post inoculation (dpi) to 48 dpi. Drawing on the protocol described by Shukla et al. (2018) and integrating the findings derived from our preliminary experiments, the acid fuchsin staining technique was adopted in the present study to stain and examine tomato roots at six distinct stages of *M. incognita* infection, which were precisely aligned with 1, 7, 14, 21, 28, and 48 days post-inoculation (dpi), respectively. Specifically, each sampling time point represents an independent infection stage, namely Stage 1 to Stage 6 in chronological order. For susceptible tomato cultivar, the interaction between tomato and *M. incognita* primarily occurs during Stages 1 to 5, and Stage 6 represents the reproductive phase of nematodes. In contrast, a certain number of nematodes can only be detected in the roots of resistant tomato cultivar during Stages 1 and 2, indicating that the tomato-nematode interaction is restricted to these two stages in resistant plants. Accordingly, Stages 1 to 5 for susceptible responses and Stages 1 to 2 for resistant responses were determined through this study.

To compare the penetration percentage of *M. incognita* between resistant and susceptible cultivars, root samples were collected at Stage 1. After gently rinsing with sterile water to remove residual soil particles, the samples were stained using the acid fuchsin staining method. The specific staining method of acid fuchsin refers to the literature by Byrd et al. (1983). Following staining, the number of J2 RKNs in the roots was observed and counted under a stereomicroscope (manufacturer: Olympus, model: SZX16). For each tomato cultivar, roots from 3 randomly selected plants were set as 3 biological replicates, and the penetration percentage was calculated using the following formula: Penetration percentage (%) = (Number of infected J2s in root/Total number of inoculated J2s) × 100%. Statistical significance of the differences in penetration percentage was determined using the Student’s *t*-test. The phenotypic photographs were captured at 48 days post inoculation of tomato plants with *M. incognita*.

### RNA extraction, library construction and sequencing

A total of 42 root samples were collected from three groups: (1) the susceptible cultivar “Jinpeng No.1” at Stages 1 to 5; (2) the resistant cultivar “Jinpeng M6” at Stage 1 and Stage 2; and (3) uninfected tomato roots of both cultivars at corresponding growth stages, which served as controls. All samples were quickly rinsed with 1× PBS to remove vermiculite around the roots, then washed thoroughly with distilled water. After freezing in liquid nitrogen for 20 minutes, the samples were used for RNA extraction. RNA was extracted using the RN40-EASYspin Plant RNA Extraction Kit (AidLab, Beijing, China). The concentration of the extracted nucleic acid was measured using a Nanodrop2000 (manufacturer: Thermo Fisher, model: Nanodrop2000), and integrity was assessed using an Agilent 2100 Bioanalyzer with LabChip GX (manufacturer: PerkinElmer, model: LabChip GX). Each sample had three biological replicates, resulting in a total of 42 samples for library construction, with 42 gene libraries constructed in total. The constructed libraries were sequenced on a BGI sequencer (model: DNBSEQ-T7). Briefly, mRNA was purified from total RNA using oligo(dT) beads, fragmented, and reverse-transcribed into cDNA with random hexamers and M-MuLV reverse transcriptase. After end pair, A-tailing, and adapter ligation, the cDNA library was size-selected, PCR-amplified, and sequenced using the PE150 strategy.

**Figure 1:**
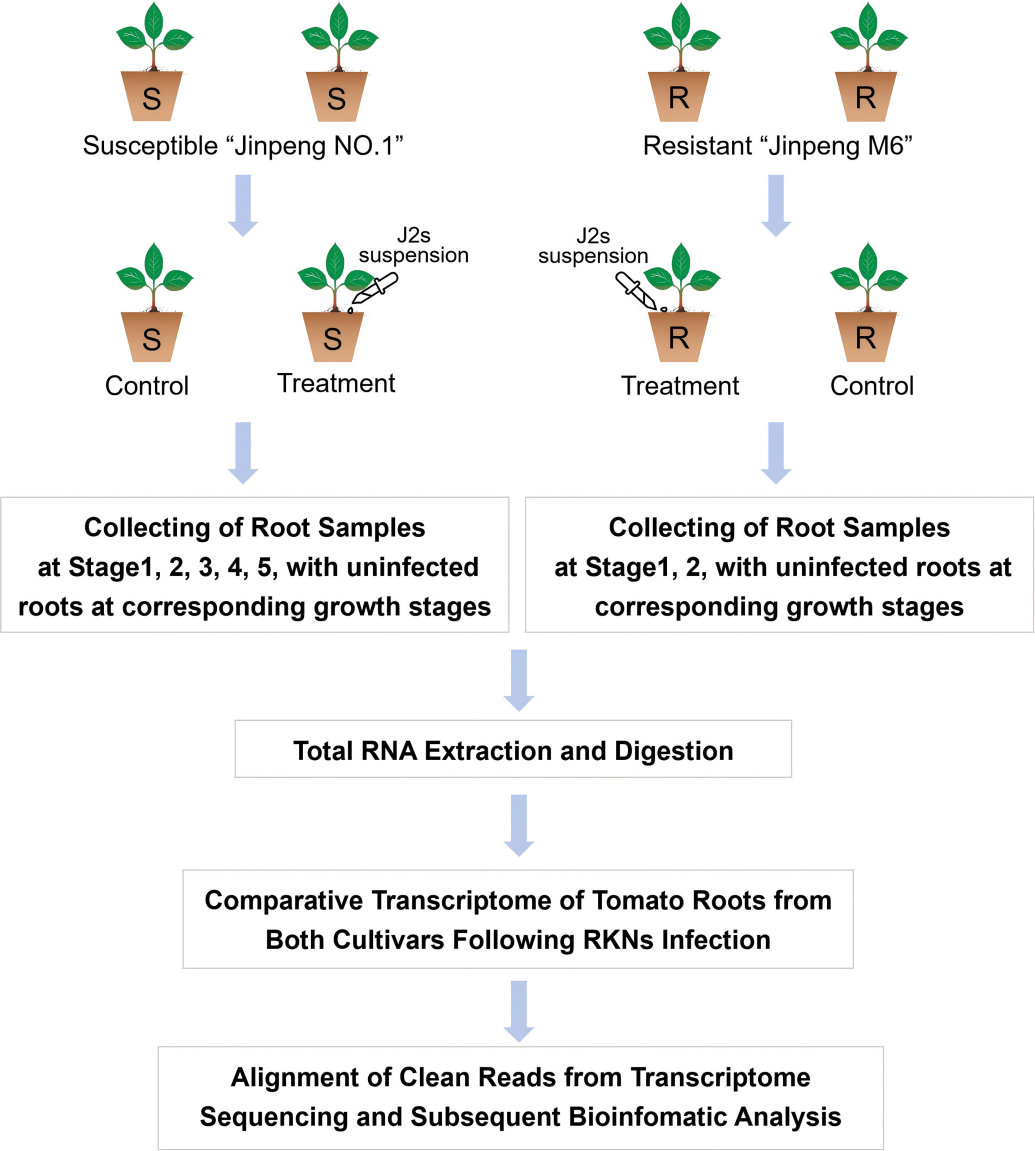
Transcriptome analysis workflow.

Clean data were obtained by filtering out adapters and low-quality reads from the raw data and then mapped to the tomato genome database using HISAT2. The mapped reads were assembled and the transcriptome was reconstructed using StringTie (Pertea et al., 2015). Htseq was used to calculate the read count values mapped to each gene, which were considered the original gene expression levels. These expressions were then normalized to fragments per kilobase of transcript per million mapped reads (FPKM). DEseq was employed for differential gene expression analysis. The screening criteria for DEGs were set as log2FoldChange > 1 and p-value < 0.05. Overall, taking tomatoes inoculated with RKN as the research object, for the susceptible tomato cultivar “Jinpeng No.1”, Stage1 to Stage5 and their corresponding uninfected control groups were set up; for the resistant tomato cultivar “Jinpeng M6”, Stage1 and Stage2 with their corresponding uninfected control groups were established. Root samples of tomatoes at each stage were collected. After total RNA extraction and enzyme digestion, dynamic transcriptome analysis of genes in tomato roots after root-knot nematode infection was carried out. Finally, relevant molecular mechanisms were explored through database retrieval and bioinformatics analysis (**Figure 1**).

### Differential expression analysis and functional enrichment

Taking tomatoes roots infected with RKN as the research object, this study analyzed the differences in gene expression levels between the susceptible tomato cultivar “Jinpeng No.1” and the resistant tomato cultivar “Jinpeng M6”. According to the relative expression levels between the susceptible and resistant cultivars and their corresponding control samples, DEGs can be divided into up-regulated genes and down-regulated genes. DEGs were screened using differential analysis software based on the gene count values of each sample, among which DESeq2 software was used for differential analysis for differential groups with biological replicates, with a threshold Log2 fold change (i.e., fold change ≥ 2 or ≤ 0.5) and a false discovery rate (FDR) < 0.05 (Love et al., 2014).

Further analysis was conducted on the DEGs, and statistical methods were employed to identify their biological functions. Gene Ontology (GO) and Kyoto Encyclopedia of Genes and Genomes (KEGG) analyses were performed to pinpoint the key biological processes or metabolic pathways involved in the defense response against RKN in both resistant and susceptible tomato cultivars. The GO annotation system is a directed acyclic graph, encompassing three major categories: Biological Process, Molecular Function, and Cellular Component. The basic unit of GO is a “term”, with each term corresponding to a specific attribute. KEGG is a database for the systematic analysis of gene functions and genomic information. It offers queries for integrated metabolic pathways, including the metabolism of carbohydrates, nucleosides, amino acids, etc., as well as the biodegradation of organic substances. It serves as a powerful tool for metabolic analysis and metabolic network research in organisms.

### Quantitative reverse transcription PCR

12 DEGs were randomly selected from the pathways significantly enriched following RKN infection in tomato. Gene-specific primers were designed using Primer Premier 5.0, targeting conserved regions of the selected DEGs. qRT PCR was performed on the Bio-Rad Real-Time PCR System (manufacturer: Bio-Rad model: CFX Connect), following the manufacturer’s instructions, with cDNA templates derived from the susceptible tomato cultivar “Jinpeng No.1” and the resistant tomato cultivar “Jinpeng M6” at multiple time points with nematode inoculation. The PCR reaction mixture (with a total volume of 20 μL) contained 1 μL of diluted cDNA template (1:10 dilution), 10 μL of 2× ChamQ Blue Universal SYBR qRT PCR Master Mix (Vazyme, Nanjing, China), and 0.4 μL of each primer (at a final concentration of 10 μM). The reactions were carried out under standard conditions: initial denaturation at 95 °C for 30 s, followed by 40 cycles of 95 °C for 10 s and 60 °C for 30 s, along with a melt curve analysis ranging from 65 °C to 95 °C. Relative gene expression levels were calculated using the 2^^(-ΔΔCt)^ method (Livak and Schmittgen, 2001), normalized against the endogenous reference gene *Actin2* (Accession ID: Solyc11g005330). Each biological replicate (n=3) consisted of independently extracted RNA samples, with three technical replicates per biological sample. For statistical analysis, data were processed using one-way analysis of variance (one-way ANOVA) in GraphPad Prism5, followed by Dunnett’s multiple comparison test to compare mean differences between multiple treatment groups and a single control group.

## Results

### Different tomato cultivars assessment of resistance to RKN

**Figure 2:**
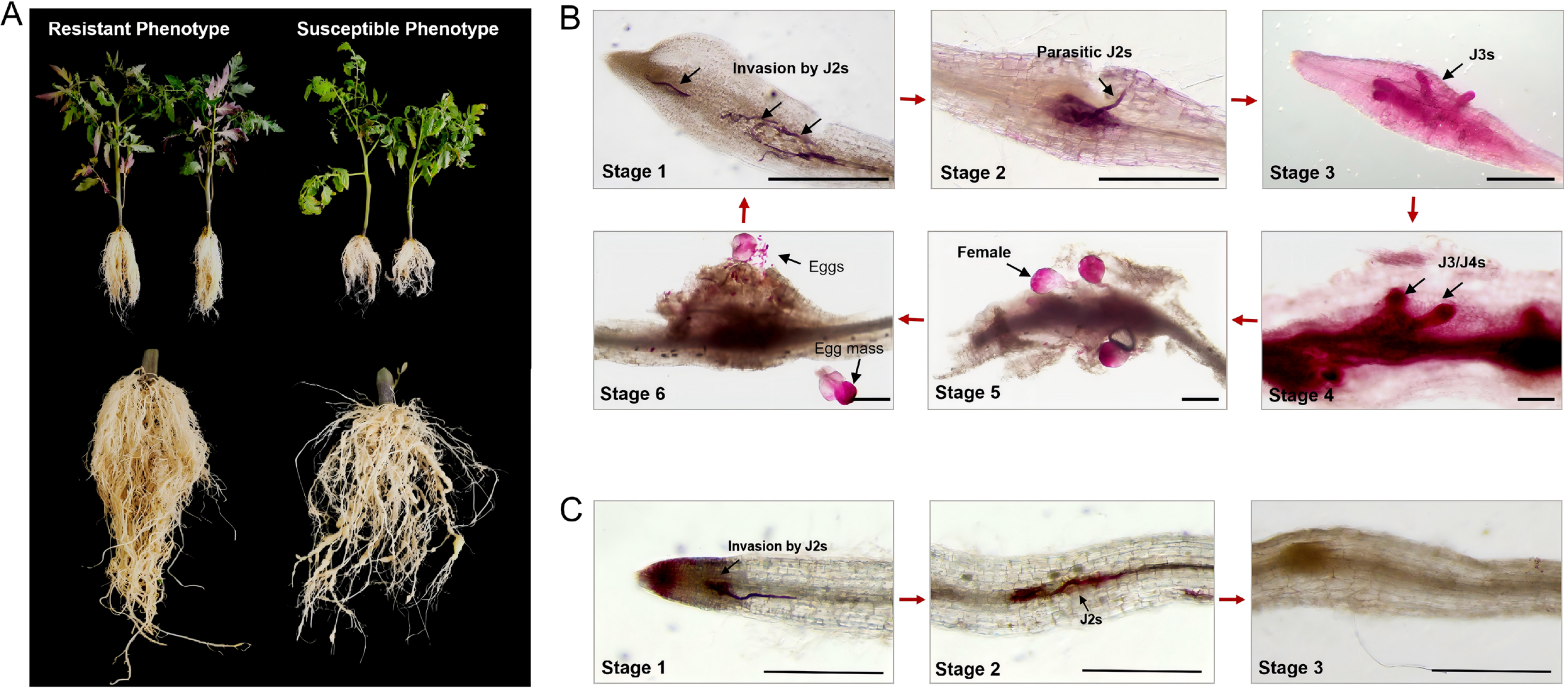
Phenotypic and histological characteristics of resistant/susceptible tomato cultivars after *M. incognita* infection. **(A)** Root phenotypes of susceptible cultivar “Jinpeng NO.1” and resistant cultivar “Jinpeng M6” after *M. incognita* infection. Photographs were captured 48 days after tomato inoculation with *M. incognita.* **(B)** Infection dynamics of *M. incognita* in susceptible tomato cultivar “Jinpeng NO.1”. **(C)** Infection dynamics of *M. incognita* in resistant tomato cultivar “Jinpeng M6”. The acid fuchsin stained tomato roots at different nematode infection stages were observed under a stereomicroscope. Bars=500 μm.

The selection of “Jinpeng No.1” and “Jinpeng M6” as subjects for this research was based on previous evaluation of various tomato cultivars’ resistance to *M. incognita*. Among these, “Jinpeng No.1” displayed high susceptibility to *M. incognita*, while “Jinpeng M6” demonstrated strong resistance (**Figure 2A**). Observations using acid fuchsin staining revealed the presence of J2 RKNs in the roots of both tomato cultivars at Stage 1 (**Figures 2B, C**). However, there was a notable difference in nematode infection levels: the penetrationpercentage in “Jinpeng No.1” reached 36%, while it was only 7% in “Jinpeng M6” (**Supplementary Figure 1**). The formation of feeding sites by nematodes in plant roots signifies the establishment of their interaction with the plants. Small root galls had formed in the roots of the susceptible tomato plants, where nematodes successfully established feeding sites and developed interactions with the plants at Stage 2 (**Figure 2B**). At Stage 3 and 4, nematodes in susceptible tomatoes roots had developed into third- and fourth-stage juveniles (J3s and J4s) (**Figure 2B**). Females matured at Stage 5, and egg masses were observable by 48 dpi (Stage 6) (**Figure 2B**). Thus, *M. incognita* completed its entire infection cycle in susceptible tomatoes within 48 days. In contrast, the nematodes in the roots of the resistant tomato plants remained in the J2 state, with their population sharply declining at Stage 2 (**Figure 2C**). As the duration of the infection increased, the nematodes in the resistant tomato roots of “Jinpeng M6” remained in the J2 state throughout the observation period and became nearly undetectable from Stage 3 to Stage 6 (**Figure 2C**). The results indicate that the interaction between “Jinpeng M6” and *M. incognita* primarily occurs during the first two developmental stages of nematode infection on tomato roots. Additionally, these findings confirm that the infection dynamics and outcomes of *M. incognita* differ significantly between susceptible and resistant tomato varieties.

### Transcriptome sequencing quality analysis

Transcriptome sequencing was performed for 5 infection stages (Stages 1 to 5) in susceptible cultivar “Jinpeng No.1”, and the first two infection stages (Stages 1 and 2) in resistant cultivar “Jinpeng M6”. This sampling design was mainly based on the distinct infection dynamics between the two cultivars in response to RKN infection (**Figure 2**). In addition, previous studies have reported that Mi-mediated resistance in tomato is an early and rapid response characterized by programmed cell death at infection sites, which restricts further interaction between nematodes and host plants (Williamson, 1998). Uninfected tomato roots at corresponding growth stages were used as controls for transcriptome sequencing analysis. Each sample was set with 3 biological replicates, and a total of 42 samples were subjected to eukaryotic reference-based transcriptome analysis. Sequencing results showed that a total of 259.74 Gb of clean data was obtained from this transcriptome sequencing, with the clean data of each individual sample not less than 5.58 Gb. The proportion of base quality value Q30 reached 95.97% and above, and the GC content of samples ranged from 41.85% to 42.77% (**Table 1**). The above data indicated that the transcriptome sequencing in this study had excellent quality and high sequence alignment efficiency, and the obtained data had high reliability. The clean reads of each sample were aligned with the tomato reference genome (Solanum_lycopersicum.ITAG2.4.genome.fa), with an average alignment efficiency of 97.83%. Based on the alignment results, alternative splicing prediction analysis, gene structure optimization analysis, and novel gene discovery were performed. A total of 2,806 novel genes were identified, among which 1,151 were functionally annotated (**Supplementary Table 1**).

**Table 1:** Statistics table of sequencing data.

Sample ID	Read Sum	Base Sum	GC(%)	N(%)	Q20(%)	Q30(%)
CK-R1-1	20557359	6153725206	42.04	0	98.87	96.98
CK-R1-2	19167876	5737878315	42.15	0	98.82	96.6
CK-R1-3	21302060	6378775352	42.16	0	98.77	96.53
CK-R2-1	21151057	6336266335	42.04	0	99.24	97.41
CK-R2-2	21453571	6419620143	42.28	0	99.23	97.35
CK-R2-3	19601040	5867613474	42.25	0	99.1	96.88
CK-S1-1	28905733	8633850000	42.17	0	99.16	97.14
CK-S1-2	19638135	5877872248	42.14	0	99.26	97.45
CK-S1-3	19912428	5961009609	42.25	0	99.04	96.67
CK-S2-1	19872308	5948188268	42.09	0	99.02	96.59
CK-S2-2	21646611	6478745957	41.96	0	98.95	96.33
CK-S2-3	19866208	5950431575	41.97	0	99.03	96.64
CK-S3-1	19592633	5869524496	41.94	0	98.97	96.39
CK-S3-2	21713554	6503593831	41.95	0	99.25	97.22
CK-S3-3	19707131	5901172843	41.85	0	99.02	96.55
CK-S4-1	19944837	5973208374	42.13	0	98.94	96.29
CK-S4-2	19786646	5927890518	42.2	0	98.83	95.97
CK-S4-3	19585621	5862955283	42.18	0	98.96	96.39
CK-S5-1	22756215	6812998137	42.14	0	98.97	96.45
CK-S5-2	20881086	6251490797	42.07	0	99.04	96.7
CK-S5-3	19999637	5988277178	42.04	0	99.02	96.6
R1-1	20490493	6134359858	42.15	0	98.85	96.88
R1-2	20564396	6158695323	42.12	0	98.8	96.68
R1-3	20468180	6127003322	42.29	0	98.86	96.87
R2-1	22171790	6639665018	42.06	0	99.25	97.42
R2-2	19985961	5985592499	42.21	0	99.02	96.6
R2-3	20030069	5995856614	42.24	0	99.07	96.79
S1-1	22652557	6779989107	42.27	0	99.05	96.7
S1-2	19871503	5949571053	42.16	0	99.07	96.77
S1-3	20355477	6096592114	42.14	0	98.97	96.37
S2-1	19704129	5900372906	41.99	0	99.03	96.67
S2-2	22846026	6841530466	42.08	0	99.01	96.34
S2-3	21843611	6541493733	42.12	0	99.09	96.65
S3-1	19899927	5958552496	42.09	0	98.97	96.44
S3-2	20818929	6235132353	42.13	0	99.07	96.76
S3-3	19409185	5811848246	42.15	0	99.04	96.62
S4-1	19494926	5828298427	42.28	0	99.13	96.97
S4-2	19315631	5784333373	42.39	0	99.07	96.71
S4-3	20644519	6184453648	42.24	0	98.96	96.37
S5-1	19673321	5893524303	41.93	0	99.16	97.12
S5-2	21800989	6482538056	42.77	0.01	98.67	96.22
S5-3	18624979	5577534797	41.99	0	98.74	96.32

### DEGs in response to RKN infection

**Figure 3:**
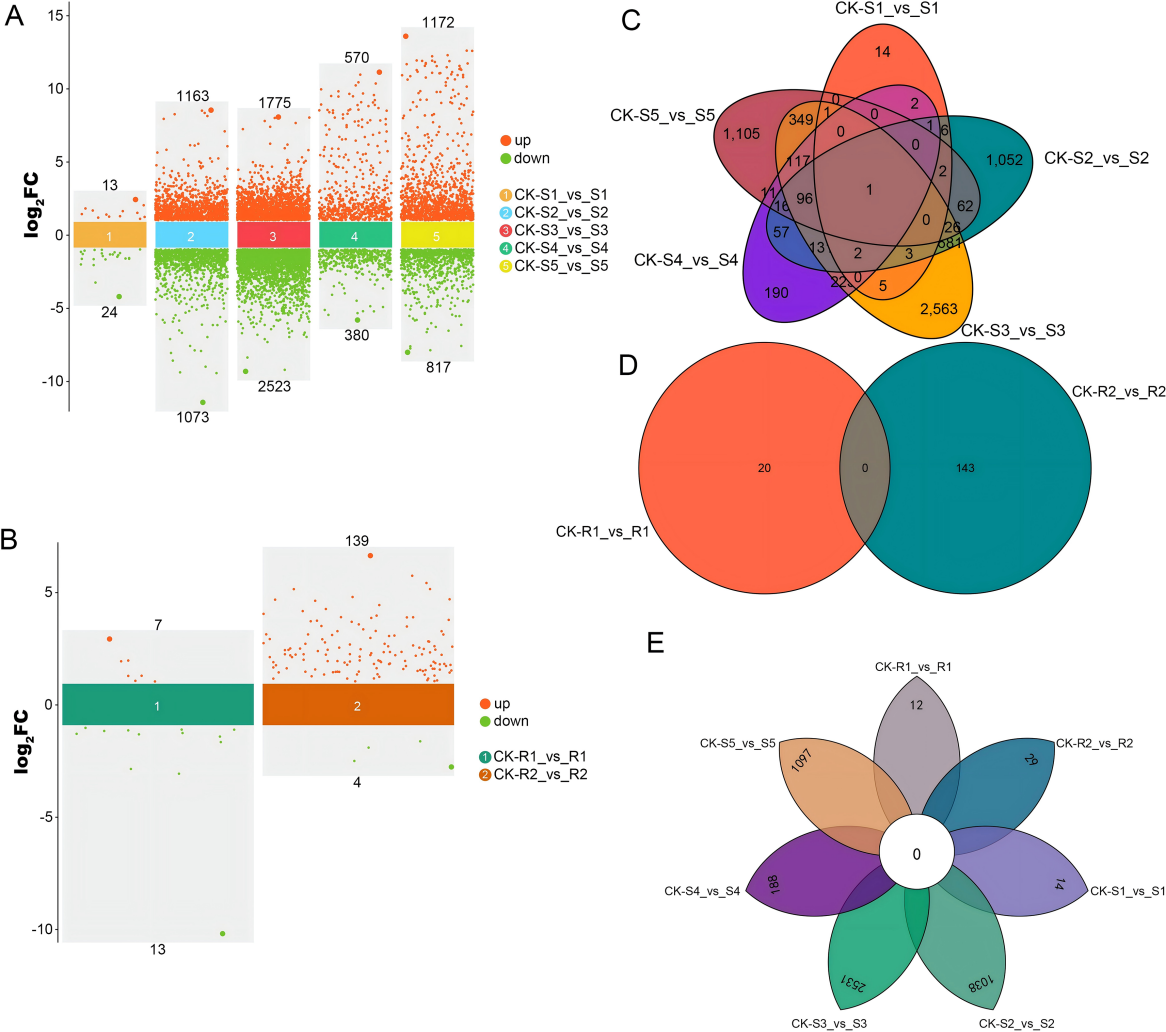
Bar chart and Venn diagram of differentially expressed genes. **(A)** Bar chart of differentially expressed genes in susceptible cultivars. **(B)** Bar chart of differentially expressed genes in resistant cultivars. **(C)** Venn diagram of differentially expressed genes in susceptible cultivars. **(D)** Venn diagram of differentially expressed genes in resistant cultivars. **(E)** Petal plot of differentially expressed genes in susceptible and resistant cultivars.

Regarding the process of *M. incognita* inoculating the susceptible tomato cultivar “Jinpeng No.1”, five infection stages, and their corresponding non-infected controls were selected to systematically analyze the disease-susceptible response. Through the stage-by-stage comparison of the roots at each inoculation stage with the controls, a total of 9,510 significantly differentially expressed genes were identified. Specifically, only 37 DEGs were found in the Stage 1, among which 13 genes were upregulated and 24 genes were downregulated. In the Stage 2, 2,236 DEGs were detected, with 1,163 genes upregulated and 1,073 genes downregulated. The number of differentially expressed genes reached its peak in the Stage 3, with a total of 4,298 DEGs, including 1,775 upregulated genes and 2,523 downregulated genes. In the Stage 4 and Stage 5, 950 DEGs (570 upregulated and 380 downregulated) and 1,989 DEGs (1,172 upregulated and 817 downregulated) were identified, respectively (**Figure 3A**).

For the resistant tomato cultivar “Jinpeng M6”, this study selected the first two inoculation stages and their corresponding controls for analysis. The results showed that compared with the non-infected controls, there were only 20 DEGs at Stage 1 (7 upregulated and 13 downregulated). The number of DEGs increased to 143 in the Stage 2, among which 139 genes were significantly upregulated and only 4 genes were downregulated. The detailed statistics of the differentially expressed genes are shown in **Table 1**. The above results indicate that after the inoculation of *M. incognita*, both “Jinpeng No.1” and “Jinpeng M6” exhibit dynamic but distinctly different gene expression response patterns at different inoculation stages (**Figure 3B**).

A Venn diagram revealed significant differences in the number of DEGs across different stages (**Supplementary Table 2**). For the susceptible tomato cultivar “Jinpeng No.1”, when comparing each of Stages 1 to 5 with their corresponding uninfected controls, there was 1 DEG common to all stages. Specifically, 14 DEGs were unique to the first stage, 1052 to the second stage, 2563 to the third stage (the largest number of unique DEGs), 190 to the fourth stage, and 1105 to the fifth stage (**Figure 3C**). For the resistant tomato cultivar “Jinpeng M6”, when comparing Stages 1 and 2 with their corresponding uninfected controls, there were no DEGs common to both stages (**Figure 3D**). When comparing the DEGs of the susceptible cultivar “Jinpeng No.1” (Stages 1–5 vs. their respective controls) with those of the resistant cultivar “Jinpeng M6” (Stages 1–2 vs. their respective controls), there were no DEGs common to all seven stages analyzed (**Figure 3E**).

### Differences in the activation of different pathways in response to RKN infection

Comparative transcriptomic analysis revealed a low number of DEGs at Stage 1 in both cultivars. At Stage 2, the number of DEGs increased markedly in both genotypes. Meanwhile, obvious phenotypic differentiation was observed in nematode morphology within different cultivar roots; roots of susceptible cultivar “Jinpeng No.1” entered the initial stage of root gall formation, whereas no root galls were detected in roots of the resistant cultivar “Jinpeng M6”. Therefore, analysis of key biological processes and metabolic pathways in response to nematode infection at Stage 2 can help clarify the pathogenic mechanism of nematodes and the differential responses between resistant and susceptible tomato cultivars.

**Figure 4:**
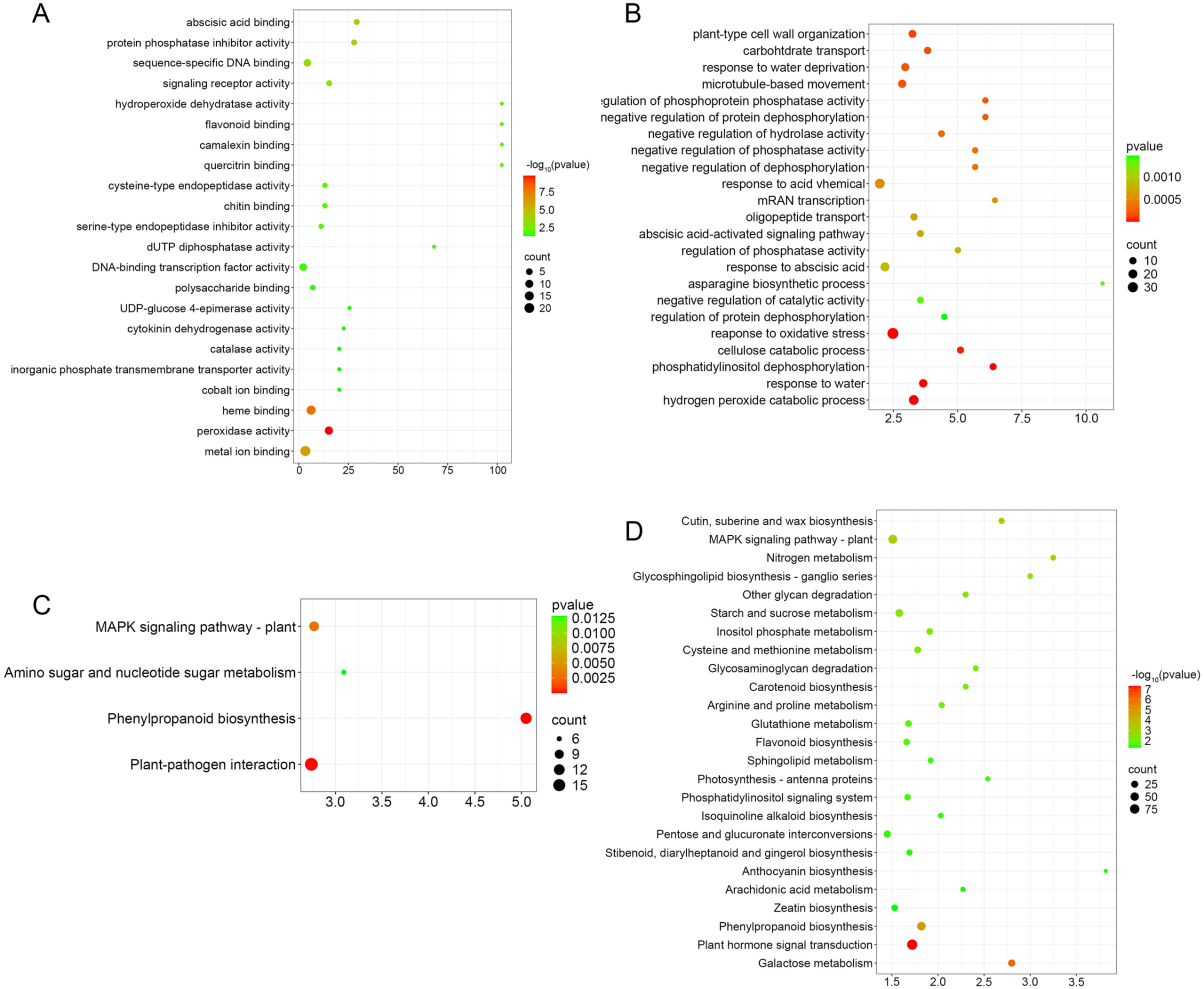
GO and KEGG analyses of differentially expressed genes at Stage 2 between the two cultivars. **(A)** GO annotation classification statistics chart of differentially expressed genes in resistant varieties. **(B)** GO annotation classification statistics chart of differentially expressed genes in susceptible varieties. **(C)** KEGG classification map of differentially expressed genes in resistant varieties. **(D)** KEGG classification map of differentially expressed genes in susceptible varieties.

To compare the functional differences of DEGs between the two cultivars, we performed GO analysis on the DEGs of the two cultivars at the Stage 2 of RKN infection to compare the GO terms of 143 DEGs in “Jinpeng M6” and 2,236 DEGs in “Jinpeng No.1”. Despite the substantial difference in the number of DEGs between “Jinpeng M6” (143 DEGs) and “Jinpeng No.1” (2,236 DEGs), the DEGs of both cultivars belong to the same categories of biological processes and molecular functions. Overall, the DEGs of “Jinpeng M6” are classified into 9 molecular functions, 15 biological processes, and 3 cellular components, while those of “Jinpeng No.1” are categorized into 11 molecular functions, 17 biological processes, and 3 cellular components. For both cultivars, the biological processes with the largest number of DEGs are “metabolic process” and “cellular process”. In terms of cellular components, the category with the most DEGs in both is “cellular anatomical entity”. For molecular functions, the DEGs with higher counts in both cultivars belong to “binding” and “catalytic activity”. In addition, “locomotion”, “rhythmic process”, “small molecule sensor activity”, and “translation regulator activity” are processes unique to the DEGs of the susceptible cultivar “Jinpeng No.1” (**Figures 4A, B**).

KEGG enrichment analysis revealed the significant pathways of DEGs in the two cultivars, with the pathways categorized into five modules: cellular processes, environmental information processing, genetic information processing, metabolism and organismal systems. The top 4 significantly enriched pathways with the smallest Q-values shared by both cultivars were “Plant MAPK signaling pathway”, “Plant hormone signal transduction”, “Phenylpropanoid biosynthesis” and “Plant-pathogen interaction”. Notably, the “Plant-pathogen interaction” pathway was the most significantly enriched in the resistant cultivar “Jinpeng M6”, accounting for 25% of its DEGs. In the susceptible tomato cultivar, the “Plant hormone signal transduction pathway” under the “Environmental information processing module” was significantly enriched, accounting for 12.71%. Additionally, the susceptible cultivar exhibited a unique pathway in the “Organismal systems module”: “Circadian rhythm - plant”, which contained 9 DEGs (1.17% of its total DEGs). Moreover, the “Phagosome process” in the “Cellular processes” module, as well as the “Phosphatidylinositol signaling system” and “ABC transporters” in the “Environmental information processing” module, were unique to the susceptible cultivar (**Figures 4C, D**).

### Genes with opposite regulation and validation of gene expression profiles by qRT-PCR

**Figure 5:**
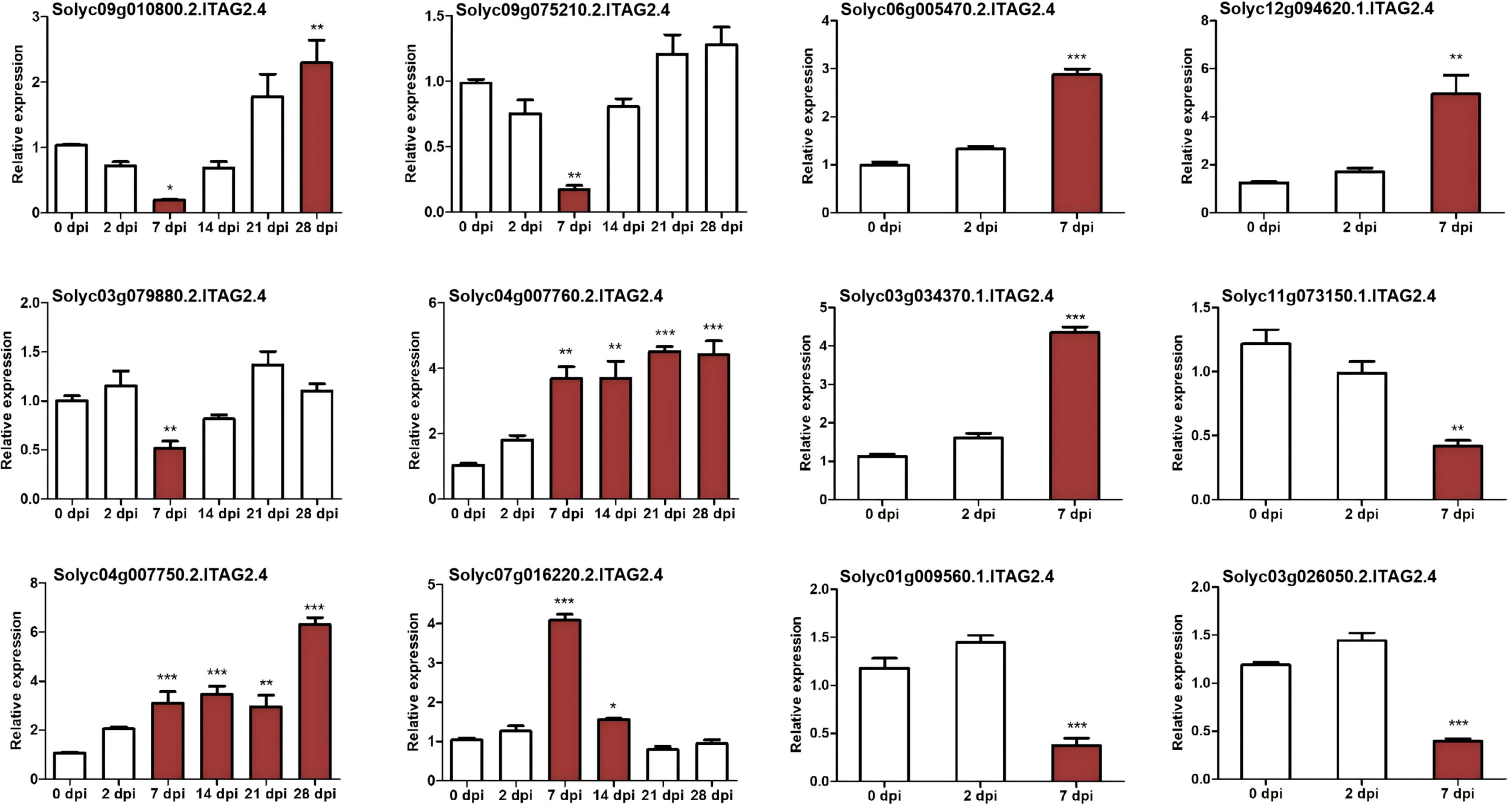
Expression analysis of selected 12 differentially expressed genes using qRT-PCR. Significance were processed by Dunnett’s multiple comparison test, and means ± SD are from three biological replicate. ^*^P ≤ 0.05, ^**^P ≤ 0.01 and ^***^P ≤ 0.001.

To screen high-quality candidate genes for subsequent functional verification, we detected 12 DEGs in resistant and susceptible tomato varieties at different infection stages using qRT-PCR technology, so as to verify the accuracy of their differential expression patterns after RKN inoculation. The results showed that although there were significant differences in the expression levels of these genes (**Figure 5**), the upregulation or downregulation trends were consistent with the RNA-seq results, indicating the reliability of the RNA-seq data. The list of qRT-PCR primer sequences can be found in **Supplementary Table 3**.

### Potential functional modules in resistant and susceptible tomato varieties

The Stage 2 after RKN infection in tomatoes corresponds to the critical period for root knot formation. At this stage, observation of the co-shared and significantly enriched “Phenylpropanoid biosynthesis” pathway in both the susceptible cultivar “Jinpeng No.1” and the resistant cultivar “Jinpeng M6” revealed substantial differences in its performance between the two cultivars at the same infection time point.

**Figure 6:**
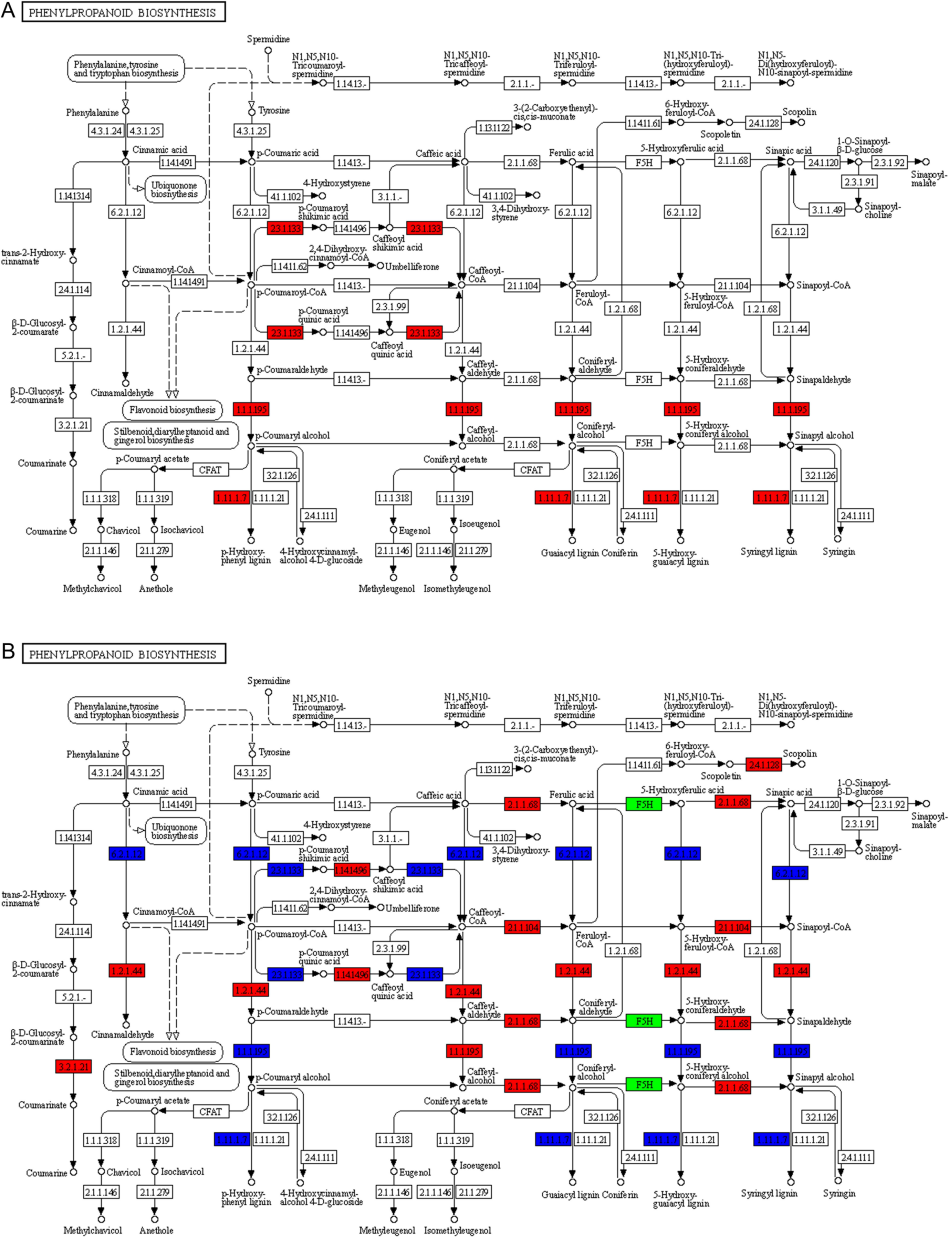
KEGG pathway annotation map of differentially expressed genes. **(A)** KEGG pathway annotation map of “Phenylpropanoid biosynthesis” for differentially expressed genes in resistant cultivars. **(B)** KEGG pathway annotation map of “Phenylpropanoid biosynthesis” for differentially expressed genes in susceptible varieties.

In the resistant tomato cultivar “Jinpeng M6”, all DEGs in this pathway were up-regulated, including “Shikimate O-hydroxycinnamoyltransferase” [EC:2.3.1.133], “Cinnamyl-alcohol dehydrogenase” [EC:1.1.1.195], and “Peroxidase” [EC:1.11.1.7] (**Figure 6A**). Nevertheless, in the susceptible tomato cultivar “Jinpeng No.1”, there were both up-regulated and down-regulated DEGs, along with a unique key enzyme: “Ferulate-5-hydroxylase” [EC:1.14.-.-]. The up-regulated genes in the resistant cultivar all showed down-regulation in the susceptible cultivar, namely “Shikimate O-hydroxycinnamoyltransferase” [EC:2.3.1.133], “Peroxidase” [EC:1.11.1.7], and the “Cinnamyl-alcohol dehydrogenase” [EC:1.1.1.195]. Additionally, the susceptible cultivar had a unique down-regulated gene: “4-Coumarate--CoA ligase” [EC:6.2.1.12] (**Figure 6B**).

The up-regulated DEGs in the susceptible cultivar included “Caffeic acid 3-O-methyltransferase” [EC:2.1.1.68], “Caffeoyl-CoA O-methyltransferase” [EC:2.1.1.104], “Cinnamoyl-CoA reductase” [EC:1.2.1.44], “β-Galactosidase” [EC:3.2.1.21], “5-O-(4-coumaroyl)-D-quinate 3’-monooxygenase” [EC:1.14.14.96], “Cinnamyl-alcohol dehydrogenase” [EC:1.1.1.195], and the “β-1,3-Galactosyltransferase” [EC:2.4.1.128]. Notably, “Cinnamyl-alcohol dehydrogenase” [EC:1.1.1.195] in the susceptible tomato cultivar exhibited both up-regulated and down-regulated expression patterns across different pathways.

## Discussion

In this study, RNA-seq was performed on samples of the resistant tomato cultivar “Jinpeng M6” and the susceptible tomato cultivar “Jinpeng No.1” at different stages after RKN infection. Quality analysis of RNA-seq data and qRT-PCR verification indicated that the RNA-seq data had good reliability, and the results derived from this data were highly authentic. This study analyzed the number and types of DEGs at different stages obtained from RNA-seq of “Jinpeng M6” and “Jinpeng No.1”. It was found that the two tomato cultivars shared no common DEGs across different stages, while the DEGs of both cultivars were enriched in the same categories of biological processes and molecular functions. We selected the Stage 2, a critical period for root knot formation after RKN infection in tomatoes, for further analysis. The results showed that there were significant differences in the number and types of DEGs between the susceptible and resistant tomato cultivars. Meanwhile, screening and analysis of physiological response modules in different tomato cultivars revealed that the Phenylpropanoid biosynthesis pathway in the ‘Metabolism’ module exhibited obvious differences between resistant and susceptible tomato cultivars. DEGs such as shikimate O-hydroxycinnamoyltransferase, peroxidase, and cinnamyl-alcohol dehydrogenase showed differences in gene expression between different cultivars. The upregulation or downregulation of these DEGs plays an important role in regulating tomato resistance or susceptibility to RKN. This study lays a foundation for subsequent research, such as selecting DEGs for gene editing to obtain disease-resistant plants and has important theoretical and practical significance for tomato disease-resistant breeding.

### Identification of DEGs from susceptible and resistant tomato cultivars

RNA-seq analysis led to the identification of 9,510 DEGs in the susceptible tomato cultivar “Jinpeng No.1” following RKN infection across five developmental stages. Notably, while only 37 DEGs were detected at Stage 1, a substantial number of DEGs were observed across Stages 2 to 5, with the highest abundance of DEGs reaching a peak at Stage 3. Nematode development analysis revealed that Stage3 represents a critical period for nematode developmental progression. During this stage, nematodes need to take up substantial amounts of nutrients from tomato cells for their larval-to-female developmental transition. Meanwhile, the massive nematode infestation disrupts the balance between defense responses and growth and development in host plant, which constitutes the primary reason for the peak number of DEGs in the host at this stage. Relevant studies have also indicated that the host transcriptional profile changes with the duration of nematode infection. Specifically, host gene expression suppression is observed in the early stages of infection, whereas the number of differentially expressed genes increases significantly in the later stages (Bar-Or et al., 2005; Jammes et al., 2005; Portillo et al., 2013). In the resistant tomato cultivar, only 163 DEGs were identified across two developmental stages, with the majority detected at Stage2. In the resistant cultivar, only a small number of nematodes can breach the root system’s physical barrier and invade the roots, which significantly weakens the interaction between the host and the nematodes. Meanwhile, we hypothesize that the defense system of resistant tomatoes precisely activates core genes directly involved in nematode suppression (e.g., key regulatory genes in the JA and SA signaling pathways).

### Differential mechanisms of susceptible and resistant tomatoes in response to RKN

In the interaction between resistant tomatoes and RKN, three cellular components showed an overall trend of up-regulation. The cellular anatomical structures play a crucial role in the ability of tomatoes to resist nematodes. The cell wall, a complex dynamic network primarily composed of cellulose, hemicellulose, and pectin, is essential in this process. When tomato plants detect nematode infection, they often disrupt the integrity of their cell walls, which triggers a series of defense responses to prevent pathogen infection (Wan et al., 2021). Significantly enriched pathways identified in the interaction between resistant tomato cultivars and RKN including JA and SA pathways, whereas no such enrichment be observed in the susceptible cultivar. The canonical SA marker gene *PR1* and *PR2* are markedly induced in the resistant cultivar but repressed in the susceptible one. Similarly, the JA marker gene *PR3* is significantly up-regulated in the resistant cultivar and down-regulated in the susceptible one, and the JA marker gene *PDF1.2* shows a stronger induction in the resistant cultivar than in the susceptible one. These results indicate that resistant and susceptible tomato cultivars in this study exhibit qualitatively distinct JA and SA signaling signatures in response to RKN infection, which is consistent with previous studies that have demonstrated the crucial roles of JA and SA pathways in plant defense against root-knot nematodes. It is reported that SlVQ15 can recruit the transcription factor SlWRKY30IIc and couple with the JA pathway to collectively regulate tomato’s immune response against RKN invasion (Huang et al., 2025). Kaempferol, an important secondary metabolite and signaling molecule in plants, is tightly regulated. The JA signaling pathway regulates kaempferol levels at multiple levels to maintain a balance between stress resistance and normal growth and development (Zhao et al., 2023). Moreover, in the protein-containing complex, proteins such as Mi-1, molecular chaperones like HSP90 and SGT1, and the SA/JA hormone pathways all play significant roles associated with Mi-1-mediated resistance (Bhattarai et al., 2007).

In susceptible tomato cultivars, the rhythmic processes within biological functions are downregulated. Plant rhythmicity is a complex regulatory network composed of genes and proteins, functioning as an intrinsic timekeeping mechanism within the plant and representing a refined adaptive strategy shaped over the course of long-term evolution. In contrast, when susceptible tomato plants are infected by nematodes, their normal biological rhythms become disrupted, which in turn leads to disturbances in processes such as the release of root exudates. This disruption impairs the plants’ ability to attract beneficial microorganisms to the rhizosphere, weakening the inhibitory effects of the microbial community on RKN and making tomato roots more susceptible to infection (Bais et al., 2006; La et al., 2024). Tomato root exudates contain chemotactic substances that attract RKN, and their release is also influenced by the biological clock. When this clock is disrupted, these substances are released in large quantities at inappropriate times, which increases the likelihood of nematodes gathering around the tomato roots, thereby raising the chances of infection (Ali et al., 2011). A noteworthy observation worth further discussion is that the susceptible cultivar uniquely enriched the “Circadian rhythm - plant” pathway in the “Organismal systems module”. Circadian rhythm is a specific rhythmic process regulated by plant endogenous circadian clock. Studies have shown that significant changes occur in plant circadian rhythm during plant-pathogen interactions (Xu et al., 2022). In the present study, such changes may stem from the massive demand for carbohydrates and amino acids by nematode-induced giant cells, whose formation disrupts the plant’s inherent diurnal metabolic rhythms including the periodicity of photosynthate transport and energy metabolism (Vanhook, 2018; de Leone et al., 2020; Mishra et al., 2022). Meanwhile, the extensive migration and feeding of a large number of nematodes within the root cells of susceptible plants cause severe cellular damage, which triggers the plant’s wound repair mechanism and induces the expression of numerous phagosome-related genes (Sobczak et al., 2011; Zou et al., 2023). This may explain the significant upregulation of genes associated with the phagosome process during the susceptible response. There is also a unique downregulation of “locomotion” within biological processes in the susceptible response. Cytoplasmic streaming in plant cells drives the movement of chloroplasts, mitochondria, and nutrients. However, due to the downregulation of locomotion, the energy supply fails to meet the demands of defense responses in a timely manner (Pandey and Dhirendra, 2018). The synthesis of defense proteins, strengthening of cell walls, and other defense mechanisms are energy-intensive processes. Inadequate energy supply weakens the defense capability of tomatoes, making them more vulnerable to nematode infestation.

### Analysis of the roles of key DEGs

The phenylpropanoid biosynthesis pathway is an important pathway for tomatoes to resist nematode infection (Zhang et al., 2015). This pathway is closely linked to the synthesis of lignin, phenolic compounds, and phytoalexins, which can form physical and chemical barriers (Liu et al., 2018). In the second stage, there are shared DEGs between the resistant tomato cultivar and the susceptible tomato cultivar, but their expression patterns differ.

Shikimate O-hydroxycinnamoyltransferase [EC:2.3.1.133] acts on the lignin synthesis branch of the phenylpropanoid pathway, catalyzing the combination of shikimate and hydroxycinnamoyl-CoA to generate hydroxycinnamoyl-shikimate ester (Adriana et al., 2021). The upregulation of this gene can accelerate the “precursor modification” step in the synthesis of lignin monomers, provide more intermediates for the subsequent production of lignin monomers, promote lignin synthesis and deposition, and strengthen the mechanical barrier of plant cell walls. The downregulation of this gene will reduce the synthesis and metabolism of hydroxycinnamate in the phenylpropanoid pathway, which may lead to insufficient supply of intermediates in the phenylpropanoid biosynthesis pathway, thereby inhibiting phenylpropanoid biosynthesis (Grover et al., 2022).

Cinnamyl-alcohol dehydrogenase [EC:1.1.1.195] is a key terminal enzyme in the lignin synthesis of the phenylpropanoid pathway. It catalyzes the reduction of cinnamaldehyde to cinnamyl alcohol, and cinnamyl alcohol is the core monomer for lignin polymerization (Shi et al., 2020; Ma et al., 2023). The upregulation of [EC:1.1.1.195] can improve the synthesis efficiency of lignin monomers, provide sufficient substrates for the polymerization of lignin in cell walls, enhance lignin content and structural stability, and help plants resist stresses such as pathogenic bacteria and mechanical damage. Its downregulation will reduce the synthesis of monolignols, lead to the obstruction of lignin synthesis, and have a negative impact on the phenylpropanoid biosynthesis pathway (Mendes et al., 2022).

Peroxidase [EC:1.11.1.7] is involved in the final polymerization stage of lignin in the phenylpropanoid pathway. It uses hydrogen peroxide (H_2_O_2_) as an oxidant to catalyze the oxidative polymerization of cinnamyl alcohol monomers, forming lignin polymers that are deposited in the cell wall (Ma et al., 2023). The upregulation of this gene can accelerate the synthesis and cross-linking of lignin polymers, enhance the rigidity and anti-degradation ability of cell walls, which act as a physical barrier to resist pathogen invasion. Meanwhile, it can also synergistically trigger plant defense responses by regulating ROS signals (Wang et al., 2024). Its downregulation will reduce its role in promoting lignin accumulation, leading to a decrease in lignin synthesis, thereby affecting the normal operation of the phenylpropanoid biosynthesis pathway.

The upregulated expression of shikimate O-hydroxycinnamoyltransferase, cinnamyl-alcohol dehydrogenase, and peroxidase forms a “relay-like” regulation. This strengthens the lignin synthesis branch of the phenylpropanoid pathway, enhances the defensive properties of plant cell walls, and helps plants cope with biotic and abiotic stresses. In contrast, the downregulated expression of these three enzymes hinders the progression of the phenylpropanoid biosynthesis pathway, leading to a significant reduction in the synthesis of phenylpropanoid compounds. This not only affects plant growth and development but also decreases their stress resistance (Liu et al., 2018; Adriana et al., 2021; Grover et al., 2022).

### Conclusion, limitations and prospects of the research

In conclusion, this study systematically analyzed the gene expression response patterns of both resistant and susceptible tomato cultivars after RKN infection. The results demonstrated marked discrepancies in the quantity and categories of DEGs between susceptible and resistant tomato cultivars. Critical DEGs were pinpointed, and the phenylpropanoid biosynthesis pathway was confirmed to be associated with tomato responses to *M. incognita* infection. This work not only lays a theoretical foundation for dissecting the molecular mechanism of tomato resistance to *M. incognita* but also furnishes a scientific rationale for the selection of resistant tomato germplasm resources. We also have limitations in the experimental design and research depth, which need to be further improved in subsequent studies. In terms of sample size, the current research only focuses on two tomato cultivars: the susceptible cultivar “Jinpeng No.1” and the resistant cultivar “Jinpeng M6”. The research results from a small number of cultivars cannot fully reflect the common response patterns of tomatoes with different genetic backgrounds to RKN infection, which affects the stability of the conclusions.

In the future, we will strengthen gene function verification and analyze the molecular mechanisms of key pathways. In the current study, a large number of DEGs were screened out through transcriptome analysis, but the specific functions of most genes remain unclear. The DEGs identified in this study from resistant and susceptible tomato cultivars, such as those encoding shikimate O-hydroxycinnamoyltransferase, peroxidase, cinnamyl-alcohol dehydrogenase, and other candidate resistance-related factors, will serve as core targets for subsequent functional research. Further studies will focus on functional validation and dissection of the molecular regulatory mechanisms of these genes to clarify their roles in tomato resistance against *M. incognita*. Meanwhile, combined with techniques such as yeast two-hybrid (Fields and Song, 1989) and luciferase assay (Gould and Subramani, 1988), the interaction between these genes and RKN effector proteins will be explored, their action targets in the infection process will be clarified, and the core molecular network of “host-nematode” interaction will be revealed. Through marker-assisted selection (MAS) technology (Gupta et al., 2009; Hasan et al., 2021), multiple disease-resistant genes will be pyramided into elite cultivars to improve the broad-spectrum resistance of the varieties. In addition, gene editing technology can be used to perform site-directed mutations on susceptibility genes in susceptible varieties, or to overexpress key defense genes in resistant varieties, so as to rapidly create new germplasms resistant to RKN. Meanwhile, combined with metabolomics analysis, the unique secondary metabolite synthesis pathways in resistant varieties will be analyzed, providing a theoretical basis for improving tomato resistance through metabolic engineering (Jia et al., 2024).

## Data Availability

All raw sequencing reads were deposited to the NCBI sequence read archive under the project ID PRJNA1354975.

## Supplementary material

Supplementary Figure 1 Penetration percentage of root-knot nematodes in resistant and susceptible tomato cultivars at Stage 1. Significance were processed by Student’s *t*-test, ***P ≤ 0.001.

Supplementary Table 1 Table of sequencing depth and alignment statistics for transcriptome sequencing samples.

Supplementary Table 2 Table of the number of differentially expressed genes across different stages.

Supplementary Table 3 Primers used in this study.
