# Supplementary figures and images for: Transcriptome-based resistance analysis of susceptible and resistant tomato cultivars against Meloidogyne incognita infection
Source: Front Plant Sci. 2026 Apr 15;17:1745106. doi: 10.3389/fpls.2026.1745106 (PMC13125003; doi:10.3389/fpls.2026.1745106)

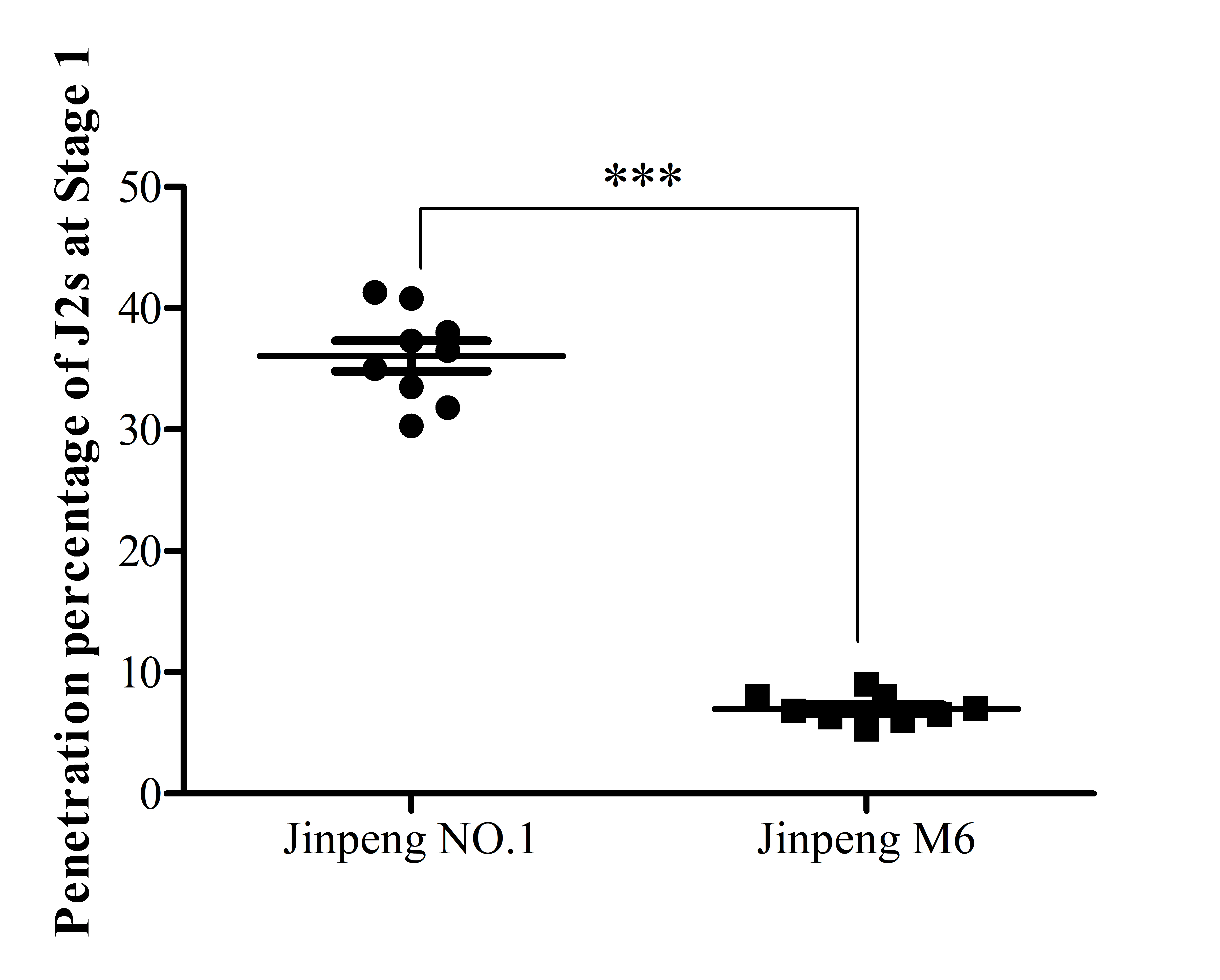

Supplement: Supplementary Figure 1 — Penetration percentage of root-knot nematodes in resistant and susceptible tomato cultivars at Stage 1. Significance were processed by Student’s t-test, ***P ≤ 0.001. [file Image1.jpeg]
